# Orientational disorder and phase transitions in crystals of (NH_4_)_2_NbOF_5_

**DOI:** 10.1107/S0108768108021289

**Published:** 2008-09-06

**Authors:** Anatoly A. Udovenko, Natalia M. Laptash

**Affiliations:** a Institute of Chemistry, Far Eastern Branch of RAS, Pr. Stoletiya 159, 690022 Vladivostok, Russian Federation

**Keywords:** ammonium oxopentafluoroniobate, distorted octahedra, dynamic orientational disorder, phase transitions, twinning, vibrational spectra

## Abstract

Ammonium oxopentafluoroniobate, (NH_4_)_2_NbOF_5_, was synthesized in a single-crystal form and the structures of its different phases were determined by X-ray diffraction at three temperatures: phase (I) at 297 K, phase (II) at 233 K and phase (III) at 198 K. The distorted [NbOF_5_]^2−^ octahedra are of similar geometry in all three structures, with the central atom shifted towards the O atom. The structure of (I) is disordered, with three spatial orientations of the [NbOF_5_]^2−^ octahedron related by a jump rotation around the pseudo-threefold local axis such that the disorder observed is of a dynamic nature. As the temperature decreases, the compound undergoes two phase transitions. The first is accompanied by full anionic ordering and partial ordering of the ammonium groups (phase II). The structure of (III) is completely ordered. The F and O atoms in the structures investigated were identified *via* the Nb—*X* (*X* = O and F) distances. The crystals of all three phases are twinned.

## Abstract 2

Structural phase transitions in a crystal of (NH_4_)_2_NbOF_5_ are the consequence of dynamic changes in its structural units as the temperature decreases. Using X-ray diffraction, it is possible to identify O and F atoms in the disordered structure of (NH_4_)_2_NbOF_5_ as a result of its dynamic nature.

## 1. Introduction

Noncentrosymmetric materials are a fertile topic of research owing to the important physical properties that may be observed in such materials: pyroelectricity, ferroelectricity, piezoelectricity or second harmonic generation (SHG). The first challenge one encounters in synthesizing a nonsymmetric material based on the [*M* ^V^OF_5_]^2−^ (*M* = V, Nb, Ta) or [*M* ^VI^O_2_F_4_]^2−^ (*M* = Mo, W) anions is to prevent oxide/fluoride ligand disorder around the transition metal. The second is to prevent these anions from crystallizing in a centrosymmetric arrangement (Marvel *et al.*, 2007 ▶). In the [NbOF_5_]^2−^ anion, out-of-center ‘primary’ electronic distortion arises from metal *d*π–oxygen *p*π orbital interactions. The Nb atom moves from the center of its coordination octahedron toward the O atom, forming a short Nb—O bond and a long *trans* Nb—F bond (Izumi *et al.*, 2005 ▶). Secondary distortions are largely dependent on anion interactions with the extended bond network. O/F ordering in a noncentro­symmetric space group has been achieved with the [NbOF_5_]^2−^ anion in inorganic–organic hybrid compounds with cluster (Heier *et al.*, 1998 ▶) and chain motifs (Norquist *et al.*, 1998 ▶). In inorganic solid-state environments, the individual Nb—O and Nb—F bonds were recently found (Marvel *et al.*, 2007 ▶) to be ordered in noncentrosymmetric KNaNbOF_5_, which exhibits the SHG property. Among the inorganic series *A*_2_NbOF_5_ [*A* = Li (Galy *et al.*, 1969 ▶), Na (Stomberg, 1984 ▶), K (Pinsker, 1966 ▶) or Cs (Fourquet *et al.*, 1973 ▶)], all compounds crystallize in centrosymmetric space groups with disordered oxide and fluoride ions.

In the present work the structures of (NH_4_)_2_NbOF_5_ at room temperature and after two phase transitions are reported, with a preference for noncentrosymmetric space group in all three cases. The compound has been known for more than 140 years and it was described by Marignac (1866 ▶) for the first time, but its structure has not been determined until now.

## 2. Experimental

### 2.1. Synthesis

(NH_4_)_2_NbOF_5_ was synthesized in a single-crystal form as colorless transparent tetrahedral prisms or polyhedra, but for the structural determination a spherical crystal was prepared. The starting materials used were of reagent grade. Niobium(V) oxide (20 g) was dissolved in 50 ml of boiling 40% hydrofluoric acid in a platinum crucible. The solution was filtered and an NH_4_F solution (NH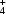:H_2_NbOF_5_ = 2.5) was added. Crystals were formed following slow evaporation in air. Analysis calculated for (NH_4_)_2_NbOF_5_: NH_4_ 15.0, Nb 38.7, F 39.6%; found: NH_4_ 15.0, Nb 38.3, F 39.4%.

The ammonia content was determined by the Kjeldahl method with a precision of ±0.3 mass%. Pyrohydrolysis at 673 K was used for simultaneous determination of the fluorine and metal content. The sample (0.2–0.4 g) was placed in a Pt boat and hydrolyzed in superheated steam for 2 h. HF was water absorbed and analyzed by titration with Th(NO_3_)_4_; the metal was analyzed gravimetrically by weighing Nb_2_O_5_. The precision of the fluorine and metal determinations was ±0.5 mass%.

### 2.2. X-ray studies

A single crystal of a spherical shape was glued to the tip of a glass needle with epoxy resin. The diffracted intensities were measured at 297 (I), 233 (II) and 198 K (III) on a Bruker SMART 1000 CCD diffractometer (Mo *K*α radiation, graphite monochromator). Scans in ω with a step size of 0.2° were performed at three ϕ settings with 2θ = −31 and −50° at a detector distance of 45 mm. Exposures of 30 s per frame were carried out in groups of 906 frames each. All reflections were indexed in the corresponding unit cells. More details on data collection and reduction are given in Table 1 ▶. Data collection, reduction and refinement of the lattice parameters were performed using *SMART* (Bruker, 1998 ▶) and *SAINT* (Bruker, 2000 ▶). All the calculations were performed with *SHELXTL* (Sheldrick, 2008 ▶). Atomic coordinates and isotropic displacement parameters for all structures are available in the deposited CIF.^1^Interatomic distances and angles are listed in Tables 2 ▶, 3 ▶ and 4 ▶ and hydrogen-bond parameters are given in Table 5 ▶.

### 2.3. Spectroscopic measurements

Mid-IR (400–4000 cm^−1^) spectra were collected in Nujol mull using a Shimadzu FTIR Prestige-21 spectrometer operating at 2 cm^−1^ resolution. FT–Raman spectra of the compound were recorded with an RFS 100/S spectrometer. The 1064 nm line of an Nd:YAG laser (130 mW maximum output) was used for excitation of the sample. The spectra were recorded at room temperature.

## 3. Results and discussion

### 3.1. Crystal structure of (I)

**Figure 1:**
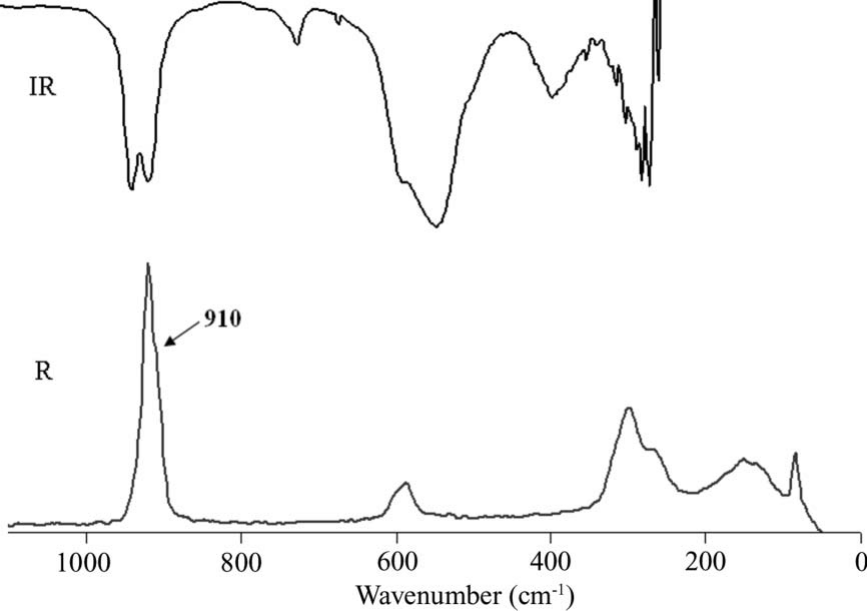
IR and Raman (R) spectra of (NH_4_)_2_NbOF_5_ at room temperature.

Structure (I) was solved, to a first approximation, by direct methods and refined against *F* ^2^ by the full-matrix least-squares method, with an anisotropic approximation to *R*_1_ = 0.0367 by location of the Nb atom in the special position (0, *Y*, *Z*) of the space group *Cmc*2_1_ (No. 36). Because of the relatively large *R*_1_ and on the basis of our preliminary ^19^F NMR data concerning the reorientation motion of [NbOF_5_] octahedra, it was suggested that the structure of (I) is disordered. Therefore, additional refinement of the structure was carried out by the displacement of the Nb atom from the special 4(*a*) position to the general 8(*b*) position; this lowered *R_1_* to 0.0316. In accordance with the vibrational spectra of (NH_4_)_2_NbOF_5_ (Fig. 1 ▶), which show two Nb states in the structure (the Nb—O stretching range contains two bands, at 933 and 912 cm^−1^ and at 920 and 910 cm^−1^ in the IR and Raman spectra, respectively), a subsequent refinement with two independent Nb atoms in special and general positions was performed; this lowered *R*1 to 0.0197. In these steps, F atoms were assigned, and then the final refinement to *R*1 = 0.0185 was made by ligand separation on O and F atoms. Atoms O1 and F1 are located on one site with different occupation parameters and equal displacement parameters, as are atoms O2 and F2. A similar procedure was used by Stomberg (1984 ▶) to discern the O and F atoms in the disordered structure of Na_2_NbOF_5_.

The occupation parameters were refined for atoms Nb1 and Nb2, and then the corresponding parameters for the F and O atoms were estimated in accordance with these refined values. The value *x* = 0.39 (5) of the Flack (1983 ▶) parameter indicated a possible twin structure of the crystal. For this reason, a final refinement was performed with the twin matrix 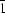
00/0
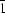
0/00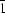, which resulted in *R*_1_ = 0.0182. The twin ratio was refined as 0.40 (3):0.60 (3) and *x* was equal to 0.0 (2).

Structure determinations of (I) were carried out in another two space groups, *Cmcm* (No. 63) and *Ama*2 (No. 40), with *R*_1_ = 0.0201 and 0.0227, respectively. It was determined that the noncentrosymmetric space group *Cmc*2_1_ was preferable because of the lower *R*_1_ value and the more reasonable Nb—*X* distances.

**Figure 2:**
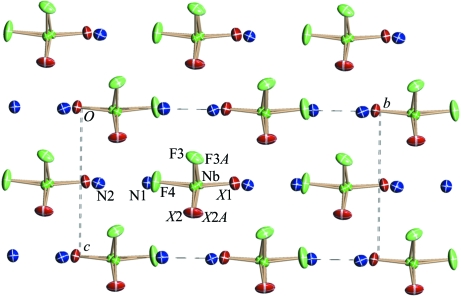
The disordered structure of (NH_4_)_2_NbOF_5_ at room temperature (I).

**Figure 3:**
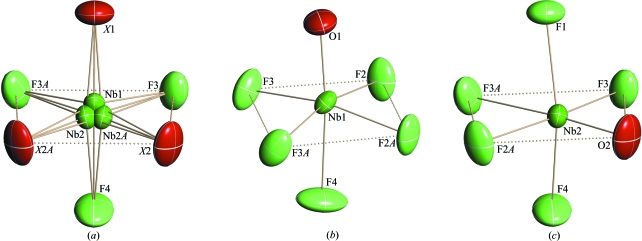
Some fragments of the structure of (I): the spatial orientations of the [NbOF_5_] octahedron (*a*); the coordination polyhedra of Nb1 (*b*) and Nb2 (*c*).

The crystal structure of (I) (Fig. 2 ▶) consists of two crystallographically independent disordered ammonium groups and disordered [NbOF_5_] octahedra in which two F atoms and one O atom occupy statistically the general (*X*2) and special (*X*1) positions (Fig. 3 ▶ *a*). The Nb atom is randomly distributed on the 4(*a*) and 8(*b*) positions, with the probabilities 0.6554 (4) and 0.1723 (2), respectively. In the [Nb1OF_5_] and [Nb2OF_5_] octahedra (Figs. 3 ▶ *b* and 3 ▶ *c*), the O atom was identified from the Nb—*X* distances (Table 2 ▶). In the Nb1 environ­ment, atom O1 occupies a special site *X*1, while it is located at the general *X*2 site in the Nb2 environment. The Nb—O distances in both polyhedra are equal to 1.733 Å, and equatorial F atoms are displaced from Nb at 1.90–1.95 Å. Niobium is shifted from the equatorial plane toward the O atom by 0.23 and 0.20 Å for Nb1 and Nb2, respectively. It should be noted that a very similar [NbOF_5_] geometry was observed in the fully ordered structures of [4-apy]_2_[Cu(4-apy)_4_(NbOF_5_)_2_] (Izumi *et al.*, 2005 ▶) and [pyH^+^]_2_[CuNb_2_(py)_4_O_2_F_10_]^2-^ (Halasyamani *et al.*, 1996 ▶). Fig. 3 ▶(*a*) shows that the [NbOF_5_] octahedra have three orientations related by local pseudo-threefold axis.

**Figure 4:**
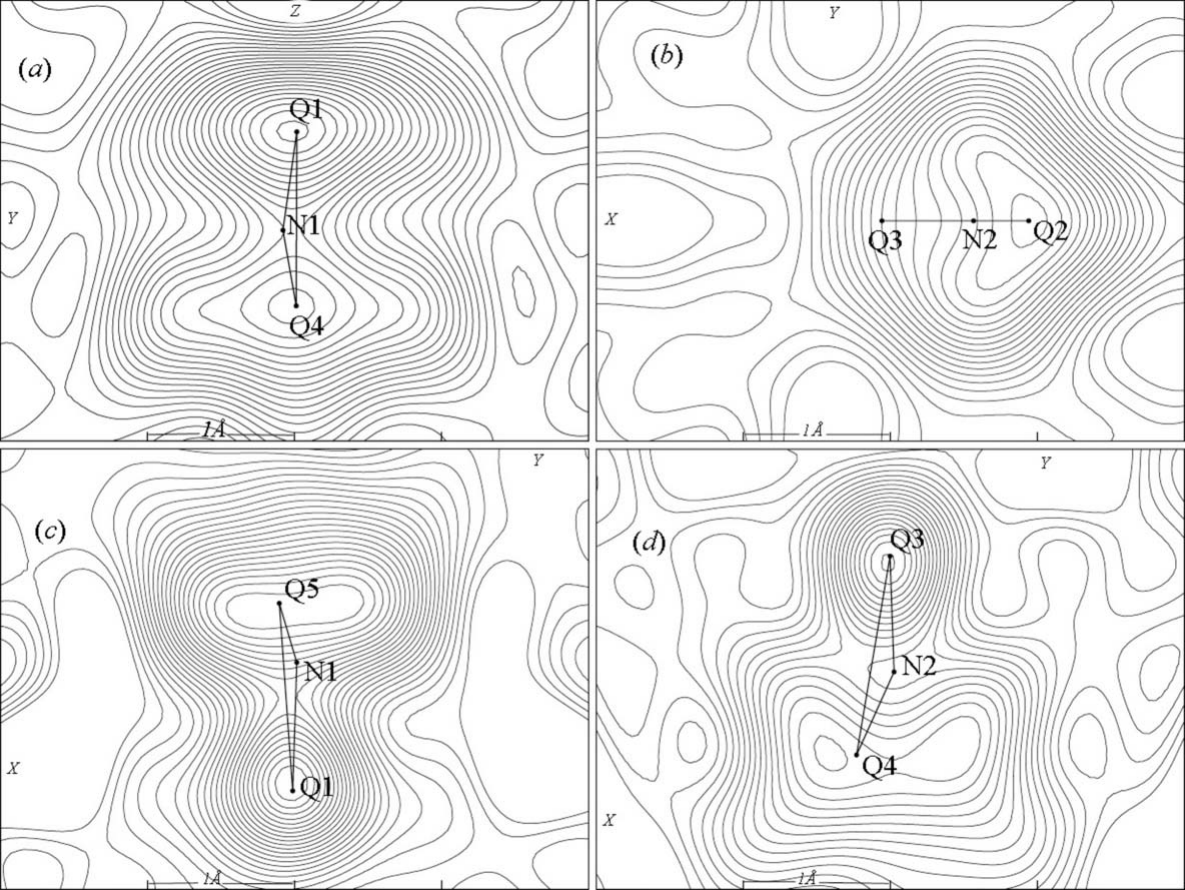
Electron difference densities around N atoms in (I) (*a*,*b*) and (II) (*c*, *d*).

H atoms in (I) are not localized. Atoms N1 and N2 are surrounded by 11 O(F) atoms in the nearest environment. The electron-density difference maps around the N atoms (Figs. 4 ▶ *a* and 4 ▶ *b*) show the hydrogen electron density to be smeared along the *c* and *a* axes for N1H_4_ and N2H_4_, respectively. Thus, the ammonium groups move in the crystal at room temperature.

### 3.2. Crystal structure of (II)

The structure of (II) was determined and refined in three monoclinic *C*-centered unit cells (*C*2, *Cm* and *C*2/*m*), which were suggested by the *BRAVAIS* and *XPREP* procedures which were used in *SMART* (Bruker, 1998 ▶) and *SAINT* (Bruker, 2000 ▶). The corresponding *R*_1_ values were 0.0193, 0.0218 and 0.0223. The Nb1—O1 and Nb2—O2 distances in the case of *Cm* are appreciably different (1.77 and 1.67 Å, respectively) – such a significant difference between these values is unacceptable. The octahedral parameters for *C*2 and *C*2/*m* are close, but the difference between two Nb—F distances and the values of four valence angles are far beyond the limits of 3σ. However, taking into account that structures (I) and (III) (see below) are noncentrosymmetric, we preferred the space group *C2* for the structure of (II). The refinement of (II) in the space group *C2* as a single crystal gave a Flack parameter of 0.57 (8), so we re-refined the structure using the twin matrix 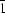
00/0
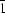
0/00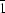 to *R*_1_ = 0.0191 with a twin ratio of 0.61 (5):0.39 (5) and *x* equal to 0.0 (3).

**Figure 5:**
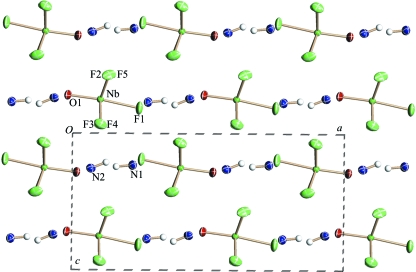
Crystal structure of (NH_4_)_2_NbOF_5_ (II) at 233 K after the first phase transition.

In the crystal structure of (II) (Fig. 5 ▶), the [NbOF_5_] polyhedra are identical and fully ordered, corresponding to a single orientational state of the anionic sublattice, *i.e.* the anions are in a static state. The octahedral geometry in (II) (Table 3 ▶) is close to that in (I). The Nb atom is displaced from the equatorial plane toward the O atom by 0.25 Å. Comparing the structures of (II) and (I), it is clear that the statistical disorder in (I) has a dynamic character. The [NbOF_5_] octahedra are in reorientational motion around the pseudo-threefold axis and form three spatial orientations in the crystal, which interchange with one another by a jump around the pseudo-threefold axis. The octahedra stop rotating during the (I) → (II) phase transition and their spatial orientations change into one orientation of the [Nb1OF_5_] octahedron. Figs. 2 ▶ and 5 ▶ show that the [NbOF_5_] polyhedra turned around the *b* axis during this process under the influence of two hydrogen bonds (Table 5 ▶).

The electron-density difference synthesis shows that only one of the H atoms is localized in each ammonium group. These atoms form hydrogen bonds with axial atoms in the octahedron (Table 5 ▶). Atoms N1 and N2 are surrounded by 12 and 9 O(F) atoms in the nearest environment, respectively. The electron-density difference maps evidence that the ammonium groups in phase (II) still rotate (Figs. 4 ▶ *c* and 4 ▶ *d*).

### 3.3. Crystal structure of (III)

We failed to solve the single-crystal structure of (III) in both triclinic and monoclinic unit cells which were determined by *BRAVAIS*. The structure was solved in the monoclinic group *Ia* (No. 9) as a two-component twin with the twin law 100/010/00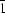 and the twin ratio 0.79 (1):0.21 (1). Without taking twinning into account, *R*_1_ was as high as 0.0529, many significant large peaks in the difference-Fourier map were observed near the Nb atoms in a difference electron density map and the H atoms were not determined. In the twin model, *R*_1_ has decreased to 0.0279 and we managed to locate all the H atoms, whereupon *R*_1_ dropped to 0.0254. However, the Flack parameter was 0.5 (1).

The centrosymmetric space group *I2*/*a* was recognized to be unsuitable for (III) since structure determination by direct methods and subsequent refinement resulted in a high *R*_1_ value of 0.134. Another refinement with initial coordinates previously obtained in *Ia* gave an *R*_1_ value of 0.0756 and highly weighted large peaks in the difference-Fourier map were observed in the electron density difference map. Thus, (III) was confirmed to be noncentro­symmetric. A series of single-crystal structure refinements was resumed in the *Ia* group, resulting in structure inversion. Final refinement with the new twin matrix 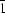
00/010/001 led to *R*_1_ = 0.0254 with a twin ratio of 0.21 (0):0.79 (0) and a Flack parameter of 0.1 (1).

**Figure 6:**
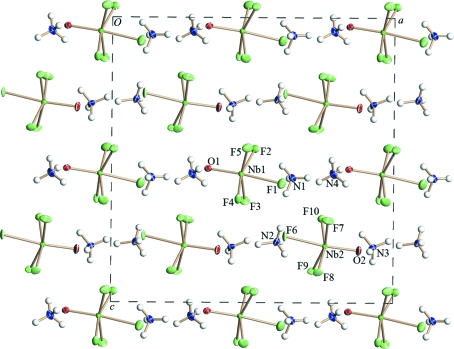
Ordered structure of (NH_4_)_2_NbOF_5_ (III) at 198 K after the second phase transition.

The crystal structure of (III) (Fig. 6 ▶) is completely ordered. It contains two types of octahedra: [Nb1OF_5_] and [Nb2OF_5_]. Their Nb—O vertices are oppositely directed along the *a* axis. Isolated octahedra are connected *via* N—H⋯O(F) hydrogen bonds (Table 5 ▶). The distances in the Nb2 octahedron are appreciably longer than those in the Nb1 octahedron (Table 4 ▶), probably as a result of the influence of hydrogen bonds. As in (I) and (II), the Nb atoms in (III) are shifted toward the O atom (by 0.26 Å). The [NbOF_5_] polyhedra are turned around the *c* axis during the (II) → (III) phase transition under the influence of hydrogen bonds (Table 5 ▶), as shown in Figs. 5 ▶ and 6 ▶.

## 4. Conclusions

It should be noted that no SHG response was observed in (NH_4_)_2_NbOF_5_, since all three structures are twinned and appreciably pseudo-centrosymmetric. The comparison of the investigated structures shows that the orientational disorder in (I) has a dynamic nature. Both niobium octahedra and ammonium tetrahedra are reoriented dynamically, so no fixed hydrogen bonds are formed. The three spatial orientations of [NbOF_5_]^2−^ around the pseudo-threefold axis arise from reorientational motion, which forces the central atom to displace from the symmetrical position and allows us to identify O and F atoms in a separate orientation of the octahedron. Thus, it becomes possible to distinguish between O and F atoms by X-ray diffraction under dynamic O/F disorder. Changes in the dynamic behavior of the complex are responsible for the phase transitions at lower temperatures.

In (II) two hydrogen bonds are formed and octahedral rotation is absent (rigid anionic sublattice), while the ammonium groups are not fully ordered. After the second phase transition [to (III)], all structural units are ordered.

## Supplementary Material

Crystal structure: contains datablocks 297K, 233K, 198K, publication_text. DOI: 10.1107/S0108768108021289/bp5012sup1.cif

Structure factors: contains datablocks 297K. DOI: 10.1107/S0108768108021289/bp5012297Ksup2.hkl

Structure factors: contains datablocks 233K. DOI: 10.1107/S0108768108021289/bp5012233Ksup3.hkl

Structure factors: contains datablocks 198K. DOI: 10.1107/S0108768108021289/bp5012198Ksup4.hkl

## Figures and Tables

**Table 1:** Crystal and experimental data for (NH_4_)_2_NbOF_5_

	297 K	233 K	198 K
Crystal data			
Chemical formula	F_5_NbO·2H_4_N	F_5_NbO·2H_4_N	F_5_NbO·2H_4_N
*M*_*r*_	239.99	239.99	239.99
Cell setting, space group	Orthorhombic, *Cmc*2_1_	Monoclinic, *C*2	Monoclinic, *Ia*
Temperature (K)	297 (2)	233 (2)	198 (2)
*a*, *b*, *c* (Å)	5.9915 (3), 14.4518 (8), 7.1999 (4)	14.4051 (9), 5.9715 (3), 7.2312 (3)	14.3384 (14), 5.9804 (6), 14.4524 (14)
β (°)	90.00	90.195 (3)	90.110 (3)
*V* (Å^3^)	623.42 (6)	622.02 (6)	1239.3 (2)
*Z*	4	4	8
*D*_*x*_ (Mg m^−3^)	2.557	2.563	2.573
Radiation type	Mo *K*α	Mo *K*α	Mo *K*α
μ (mm^−1^)	1.97	1.97	1.98
Crystal form, color	Sphere, colorless	Sphere, colorless	Sphere, colorless
Crystal size (mm)	0.32 × 0.32 × 0.32	0.32 × 0.32 × 0.32	0.32 × 0.32 × 0.32
			
Data collection			
Diffractometer	Bruker SMART 1000 CCD	Bruker SMART 1000 CCD	Bruker SMART 1000 CCD
Data collection method	ω scans	ω scans	ϕ and ω scans
Absorption correction	Multi-scan	Multi-scan	Multi-scan
*T*_min_	0.572	0.571	0.570
*T*_max_	0.572	0.571	0.570
No. of measured, independent and observed reflections	8132, 1881, 1806	8132, 3062, 3003	16 431, 6354, 5715
Criterion for observed reflections	*I* > 2σ(*I*)	*I* > 2σ(*I*)	*I* > 2σ(*I*)
*R*_int_	0.028	0.025	0.025
θ_max_ (°)	39.0	39.0	39.0
			
Refinement			
Refinement on	*F*^2^	*F*^2^	*F*^2^
*R*[*F*^2^ > 2σ(*F*^2^)], *wR*(*F*^2^), *S*	0.017, 0.049, 1.08	0.019, 0.052, 1.14	0.023, 0.057, 1.05
No. of reflections	1881	3062	6354
No. of parameters	61	84	165
H-atom treatment	Not refined	Not refined	Not refined
Weighting scheme	*w* = 1/[σ^2^(*F* 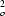 ) + (0.0301*P*)^2^ + 0.0923*P*], where *P* = (*F* 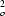 + 2*F* 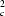 )/3	*w* = 1/[σ^2^(*F* 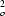 ) + (0.0261*P*)^2^ + 0.3566*P*], where *P* = (*F* 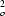 + 2*F* 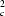 )/3	*w* = 1/[σ^2^(*F* 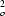 ) + (0.0199*P*)^2^ + 1.2051*P*], where *P* = (*F* 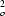 + 2*F* 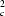 )/3
(Δ/σ)_max_	0.040	0.020	0.108
Δρ_max_, Δρ_min_ (e Å^−3^)	0.57, −0.41	0.99, −0.97	0.89, −1.28
Extinction method	*SHELXL*97	*SHELXL*97	*SHELXL*97
Extinction coefficient	0.1814 (17)	0.1807 (13)	0.02053 (17)
Absolute structure	Flack (1983 ▶)	Flack (1983 ▶)	Flack (1983 ▶)
Flack parameter	0.0 (2)	0.0 (3)	0.11 (10)

Computer programs used: *SMART* (Bruker, 1998 ▶), *SAINT* (Bruker, 2000 ▶), *SHELXTL* (Sheldrick, 2008 ▶).

**Table 2:** Selected distances (Å) and angles (°) for (I)

Nb1—O1	1.734 (1)	Nb2—F4	1.904 (1)
Nb1—F4	2.089 (1)	*X*1—*X*2†	2.805 (1) ×2
Nb1—F2	1.945 (1) ×2	*X*1—F3	2.697 (2) ×2
Nb1—F3	1.933 (1) ×2	F4—*X*2	2.722 (2) ×2
Nb2—O2	1.735 (1)	F4—F3	2.635 (2) ×2
Nb2—F3*A*	2.116 (1)	F2—F2*A*	2.702 (2)
Nb2—F1	1.952 (1)	F2—F3	2.665 (1) ×2
Nb2—F2*A*	1.944 (1)	F3—F3*A*	2.855 (2)
Nb2—F3	1.915 (1)		
			
O1—Nb1—F2	99.18 (6) ×2	F3*A*—Nb2—F3	89.93 (2)
O1—Nb1—F3	94.52 (5) ×2	F3*A*—Nb2—F4	81.56 (2)
F4—Nb1—F2	84.77 (6) ×2	F1—Nb2—F3	88.49 (2)
O2—Nb2—F1	98.90 (6)	F3—Nb2—F4	87.27 (7)
O2—Nb2—F2*A*	94.33 (6)	F3—Nb1—F3*A*	95.20 (6)
O2—Nb2—F3	93.67 (4)	O1—Nb1—F4	174.46 (9)
O2—Nb2—F4	96.70 (7)	F3—Nb1—F2*A*	165.96 (4) ×2
F3*A*—Nb2—F1	82.93 (5)	F4—Nb2—F2*A*	90.01 (6)
F4—Nb1—F3	81.64 (2) ×2	F2*A*—Nb2—F1	92.10 (5)
F2—Nb1—F2*A*	87.96 (2)	O2—Nb2—F3*A*	175.91 (5)
F2—Nb1—F3	86.82 (2) ×2	F2*A*—Nb2—F3	171.80 (5)
F3*A*—Nb2—F2*A*	81.88 (2)	F1—Nb2—F4	164.05 (8)

† *X* = F(O).

**Table 3:** Selected distances (Å) and angles (°) for (II)

Nb1—O1	1.727 (1)	O1—F2	2.797 (2)	F1—F4	2.569 (2)
Nb1—F1	2.122 (1)	O1—F3	2.762 (2)	F1—F5	2.691 (2)
Nb1—F2	1.948 (1)	O1—F4	2.701 (2)	F2—F3	2.634 (2)
Nb1—F3	1.959 (1)	O1—F5	2.785 (2)	F3—F4	2.804 (1)
Nb1—F4	1.917 (1)	F1—F2	2.797 (2)	F4—F5	2.712 (1)
Nb1—F5	1.945 (1)	F1—F3	2.689 (2)	F5—F2	2.742 (1)
					
O1—Nb1—F2	98.9 (1)	F1—Nb1—F2	86.7 (1)	F2—Nb1—F3	84.8 (1)
O1—Nb1—F3	96.8 (1)	F1—Nb1—F3	82.3 (1)	F3—Nb1—F4	92.7 (1)
O1—Nb1—F4	95.5 (1)	F1—Nb1—F4	78.8 (1)	F4—Nb1—F5	89.2 (1)
O1—Nb1—F5	98.5 (1)	F1—Nb1—F5	82.7 (1)	F5—Nb1—F2	89.5 (1)
O1—Nb1—F1	174.2 (1)	F2—Nb1—F4	165.5 (1)	F3—Nb1—F5	164.3 (1)

**Table 4:** Selected distances (Å) and angles (°) for (III)

Nb1—O1	1.721 (1)	O1—F2	2.801 (2)	F1—F4	2.620 (2)
Nb1—F3	1.912 (1)	O1—F3	2.715 (2)	F1—F5	2.730 (2)
Nb1—F4	1.929 (1)	O1—F4	2.722 (2)	F2—F3	2.676 (2)
Nb1—F2	1.934 (1)	O1—F5	2.778 (2)	F3—F4	2.789 (2)
Nb1—F5	1.943 (1)	F1—F2	2.719 (2)	F4—F5	2.638 (2)
Nb1—F1	2.134 (1)	F1—F3	2.636 (2)	F5—F2	2.707 (2)
Nb2—O2	1.737 (1)	O2—F7	2.756 (2)	F6—F9	2.777 (2)
Nb2—F10	1.949 (1)	O2—F8	2.839 (2)	F6—F10	2.623 (2)
Nb2—F7	1.953 (1)	O2—F9	2.792 (2)	F7—F8	2.663 (2)
Nb2—F8	1.954 (1)	O2—F10	2.732 (2)	F8—F9	2.758 (2)
Nb2—F9	1.956 (1)	F6—F7	2.639 (2)	F9—F10	2.695 (2)
Nb2—F6	2.134 (1)	F6—F8	2.748 (2)	F10—F7	2.832 (2)
					
O1—Nb1—F2	99.90 (5)	F1—Nb1—F2	83.76 (5)	F2—Nb1—F3	88.17 (5)
O1—Nb1—F3	96.56 (5)	F1—Nb1—F3	81.13 (5)	F3—Nb1—F4	93.13 (5)
O1—Nb1—F4	96.32 (5)	F1—Nb1—F4	80.17 (5)	F4—Nb1—F5	85.91 (5)
O1—Nb1—F5	98.44 (6)	F1—Nb1—F5	83.95 (6)	F5—Nb1—F2	88.59 (5)
O1—Nb1—F1	175.64 (5)	F2—Nb1—F4	163.47 (5)	F3—Nb1—F5	164.99 (6)
O2—Nb2—F7	96.46 (7)	F6—Nb2—F7	80.31 (6)	F7—Nb2—F8	85.94 (5)
O2—Nb2—F8	100.41 (7)	F6—Nb2—F8	84.34 (5)	F8—Nb2—F9	89.73 (5)
O2—Nb2—F9	98.02 (7)	F6—Nb2—F9	85.41 (5)	F9—Nb2—F10	87.29 (5)
O2—Nb2—F10	95.51 (6)	F6—Nb2—F10	79.82 (5)	F10—Nb2—F7	93.07 (5)
O2—Nb2—F6	174.11 (7)	F7—Nb2—F9	165.41 (7)	F8—Nb2—F10	164.07 (6)

**Table 5:** Hydrogen-bond parameters (Å, °) in (II) and (III)

*D*—H⋯*A*	*D*—H	H⋯*A*	*D*⋯*A*	*D*—H⋯*A*
(II)				
N1—H1⋯O1	0.88	2.12	2.997 (1)	177
N2—H2⋯F1	0.86	1.89	2.721 (1)	164
				
(III)				
N1—H1⋯O1^i^	0.80	2.15	2.921 (2)	161
N1—H2⋯F2^ii^	0.90	2.04	2.908 (2)	162
N1—H3⋯F6	0.91	2.16	2.988 (2)	151
N1—H4⋯F1	0.80	2.01	2.806 (2)	171
				
N2—H5⋯F9	0.84	2.07	2.862 (1)	158
N2—H6⋯F1	0.80	2.27	3.000 (1)	150
N2—H7⋯O2^iii^	0.94	2.08	2.986 (1)	174
N2—H8⋯F6^iv^	0.88	1.93	2.806 (1)	170
				
N3—H9⋯F6^i^	0.87	1.91	2.762 (2)	165
N3—H10⋯F7^iv^	0.89	2.22	3.030 (2)	151
N3—H11⋯O2	0.81	2.11	2.896 (2)	163
N3—H12⋯F5^v^	0.87	1.98	2.810 (2)	157
				
N4—H13⋯F4^i^	0.87	2.18	3.036 (2)	168
N4—H14⋯F8^vi^	0.84	2.02	2.835 (2)	166
N4—H15⋯F1	0.84	1.94	2.683 (2)	148
N4—H16⋯O1^vii^	0.87	2.12	2.961 (2)	162

Symmetry codes: (i) 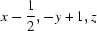; (ii) 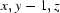; (iii) 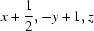; (iv) 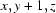; (v) 
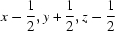
; (vi) 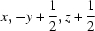; (vii) 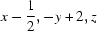.

## Footnotes

1 Supplementary data for this paper are available from the IUCr electronic archives (Reference: BP5012). Services for accessing these data are described at the back of the journal.
